# Cellular phenotype database: a repository for systems microscopy data

**DOI:** 10.1093/bioinformatics/btv199

**Published:** 2015-04-09

**Authors:** Catherine Kirsanova, Alvis Brazma, Gabriella Rustici, Ugis Sarkans

**Affiliations:** European Molecular Biology Laboratory, European Bioinformatics Institute (EMBL-EBI), Wellcome Trust Genome Campus, Hinxton CB10 1SD, UK

## Abstract

**Motivation:** The Cellular Phenotype Database (CPD) is a repository for data derived from high-throughput systems microscopy studies. The aims of this resource are: (i) to provide easy access to cellular phenotype and molecular localization data for the broader research community; (ii) to facilitate integration of independent phenotypic studies by means of data aggregation techniques, including use of an ontology and (iii) to facilitate development of analytical methods in this field.

**Results:** In this article we present CPD, its data structure and user interface, propose a minimal set of information describing RNA interference experiments, and suggest a generic schema for management and aggregation of outputs from phenotypic or molecular localization experiments. The database has a flexible structure for management of data from heterogeneous sources of systems microscopy experimental outputs generated by a variety of protocols and technologies and can be queried by gene, reagent, gene attribute, study keywords, phenotype or ontology terms.

**Availability and implementation:** CPD is developed as part of the Systems Microscopy Network of Excellence and is accessible at http://www.ebi.ac.uk/fg/sym.

**Contact:** jes@ebi.ac.uk or ugis@ebi.ac.uk

**Supplementary information:** Supplementary data are available at *Bioinformatics* online.

## 1 Introduction

A novel ‘systems microscopy’ research strategy has emerged during the last decade that exploits recent developments in automated microscopy, cell microarrays, image analysis, data mining, statistics and modelling (Lock *et* *al.*, 2010). A wide range of methodologies is available to the scientific community for the characterization of cellular processes like mitosis or cell migration. For example, RNA interference (RNAi) screening approaches deliver diverse sets of cell loss-of-function phenotypes, assigned manually or automatically using various methodologies and processing pipelines.

As part of the Systems Microscopy Network of Excellence project, we have developed the Cellular Phenotype Database (CPD) for management of data derived from high-throughput phenotypic studies. Reference studies like Mitocheck (Neumann *et* *al.*, 2003) and CellMorph (Fuchs *et* *al.*, 2010) provided use cases and initial datasets for CPD development. In total, data from 10 studies have been loaded into the database (Di *et* *al.*, 2012; Gudjonsson *et* *al.*, 2012; Moudry *et al.*, 2011; Ritzerfeld *et* *al.*, 2011; Rohn *et* *al.*, 2011; Schmitz *et* *al.*, 2010; Simpson *et* *al.*, 2012). The database has been released to the public.

Across these studies we have observed high phenotypic heterogeneity both in terms of the criteria used to assign phenotypes (via manual or automatic annotation), and the terminology used for phenotypic annotations. Phenotypes observed can be specific to a given study or common across several studies. In the latter case terms used to describe phenotypes can be harmonized via a common ontology. To achieve this, the Cellular Microscopy Phenotype Ontology (CMPO; Jupp *et* *al.*, 2014) was used by appending CMPO terms to the original phenotype annotation, and ontology based browsing was implemented in the CPD user interface.

We propose a minimal set of descriptors for reporting RNAi studies, and describe the data representation and exploration approaches we developed that can potentially facilitate observation of patterns emerging from a variety of data acquisition methods and protocols. Although currently in CPD we have integrated datasets from RNAi-based high-throughput phenotypic studies only, the same data management methodology could be used for aggregation of data generated in other types of systems microscopy experiments.

## 2 Generic data structure

All information stored in the database, and represented through the web-interface, can be categorized into three layers:

1. Input data layer:
  - study description: general information, specific screen information (including protocols), list of phenotypes or features observed, and definitions that enable interpretation of submitter-specific results output (see next point; details in the Implementation section);
  - processed submitter’s pipeline outputs, such as reagent identification and quantitative scores;
  - genome data with coding transcript sequences and Gene Ontology annotation; and
  - siRNA reagent data with sequences (library files).
2. Aggregated data layer derived from input data:
  - processed data objects, where each object stores data about genes and reagents that map to a given phenotype set; and
  - ontology mappings to the original phenotype terms.
3. Web interface layer:
  - data object pages (Gene, Reagent, Replica, Study); and
  - data aggregation pages representing ‘gene-phenotype’ relationships.

Aggregated data layer could also store information about intracellular compartments, i.e. which genes and reagents are related to which intracellular compartment or other molecular localization objects—see also the Section 6.

**Fig. 1.**
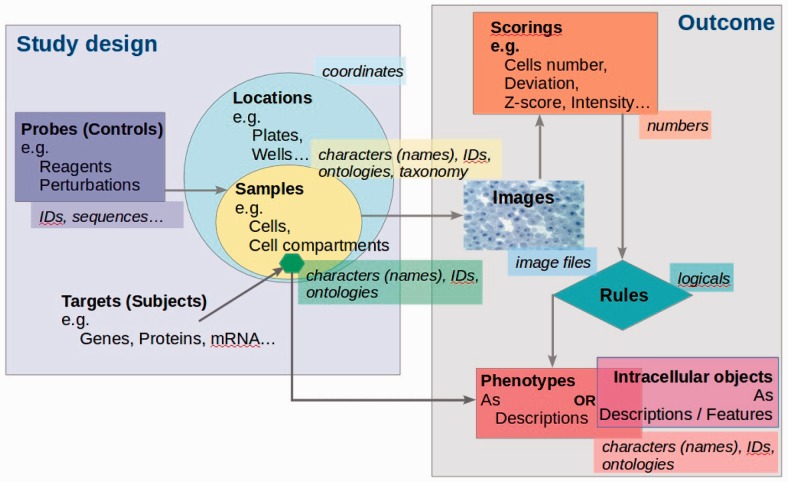
Schematic representation of studies suitable for CPD

The overall schema for the studies which can be loaded into CPD is represented in Figure 1. If a given study design and outcome comply with this schema, then the study data can be processed and loaded into the database. Additional metadata about the study, its protocols and scoring methods, including phenotype definitions, are structured during the data curation stage to conform to the Study Description File (SDF) specifications—see the ‘Implementation’ section below. Each new study that is to be loaded into the database is analyzed for compatibility with SDF. A new study type can be introduced by producing a new SDF template that defines how outputs of such studies can be processed when loading into the database; the SDF templates are stored as top level objects in the database.

## 3 Minimum reporting requirements

We suggest that the following information is essential when sharing phenotypic data derived from RNAi-based studies in order to make it discoverable, reusable and interpretable:

1. Generic information about a study including title, description, publication details (if applicable), authors contact details and the number of screens included in the study;
2. Information specific to each screen in the study, including target organism, materials used (e.g. cell line), reagent library details (e.g. manufacturer, reagent type, library version), names and descriptions of the experimental and analytical protocols used, list of all phenotype terms used in the study, logical scoring rules for phenotype calculation (unless phenotypes have been directly assigned), descriptions of the phenotypes observed, all phenotypes scoring parameters and their types, processed data file structure and description (including data column names, positions and types), see (4);
3. The reagent library annotation, including, as the minimum, reagent IDs and sequences;
4. The processed data file containing the screen results in the form of a table where each row corresponds to a plate position, and therefore to a replica with particular reagent. Additional columns must contain the score(s) that has been used to assign a phenotype to that particular position/reagent and the phenotype term(s) if they are directly provided, otherwise phenotype definition rules defined in (2) are to be applied. In special cases when images are also to be submitted, for each plate position in the processed data file we require an additional column with the full image path for the image(s) associated with the selected position/reagent. This column can be split into several columns (one per channel), if needed.

Previous efforts for reporting minimum information about an RNAi experiment exist (http://miare.sourceforge.net/HomePage). Our suggested requirements are similar to those guidelines for capturing the basic RNAi experiment metadata; additionally, we propose here requirements for capturing phenotype details.

The above information is processed by the CPD submission pipeline and may be updated with information from any other phenotypic or molecular localization study in a similar way by generating a corresponding SDF template—see the Section 5 below.

## 4 User interface

The following requirements were taken into account during user interface development:

1. the interface should be intuitive and easy to use;
2. response times should be fast;
3. users should be able to access all information available;
4. data browsing functionality should be available to help users who might be unsure about what search term to use; and
5. filtering options should be available for large query results.

Through the CPD interface, users can search for genes of interest by gene symbols, Ensembl identifiers, or attributes (e.g. Gene Ontology terms), and retrieve the loss-of-function phenotypes observed by suppressing the expression of the selected gene(s), through RNAi reagents, across independent phenotypic studies.

Similarly, users can search for a phenotype of interest and retrieve the RNAi reagents that have caused such phenotype(s) and the associated target genes. Search for a phenotype, or a set of phenotypes, is supported either by the original phenotypic annotations provided by the submitters, or by the corresponding CMPO ontology term(s), which are added at the curation stage. The reagents that have caused such phenotype(s) are displayed, together with the associated target gene. Information about specific reagents can be obtained when querying by a reagent identifier. Users can also explore datasets by browsing phenotypes, either by the original phenotypic annotation or by CMPO terms.

Genes and phenotypes query functionalities are enriched by an autocomplete plugin, which limits users to enter only gene symbols, synonyms, Ensembl identifiers, phenotype(s) or CMPO term(s) which are already present in CPD. Alternatively, users can explore datasets by searching studies by keyword.

**Fig. 2.**
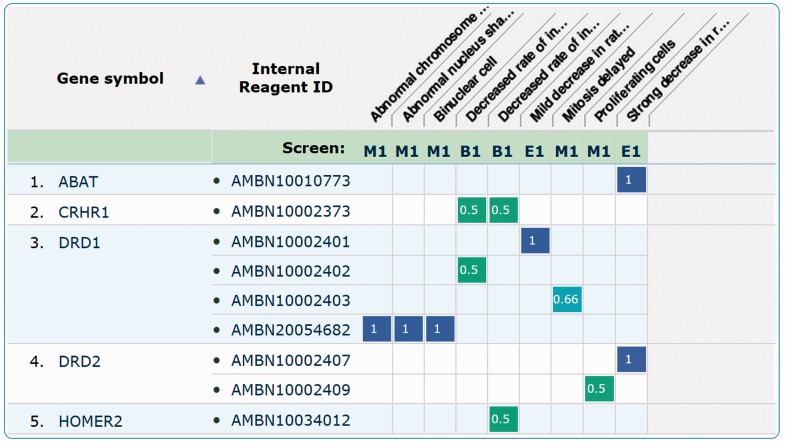
Example of database search result page in response to a query by gene attribute (GO term). Five genes are retrieved (e.g. DRD2), alongside the reagents (e.g. AMBN20054682) mapping to them and the associated phenotypes (e.g. Binuclear cell). The ‘Study’ row lists different study acronyms where phenotypes (here represented by CMPO terms) have been observed. For each gene-reagent-phenotype triplet, a measure of the reagents reproducibility is given. Please note that these scores are not comparable across independent studies but only within each study

Datasets can be retrieved and visualized so that users can compare the observed phenotypes within a given study or across independent studies. The latter option allows them to cross-correlate phenotypic information obtained in different studies where the same or different RNAi libraries have been used. The results of querying the database by gene attribute(s), phenotype(s) or CMPO term(s) are represented by a ‘genes-to-phenotypes’ heatmap (Fig. 2), where the numerical value and the corresponding colour in a heatmap cell represents evidence levels of the reagent reproducibility within a study, across different replicas. Heatmap cells are hyperlinked to the ‘replica page’ specific for the study scoring methods.

Users can also browse data by phenotype or CMPO ontology terms. When browsing, multiple phenotypes, either from the same or different studies, can be selected to retrieve the list of genes (reagents) where interference resulted in all of the selected phenotypes. The results are again presented as a heatmap.

Each study has a dedicated page displaying the associated metadata and linking to direct download of the associated data available, for each screen in the selected study.

## 5 Implementation

**Fig. 3.**
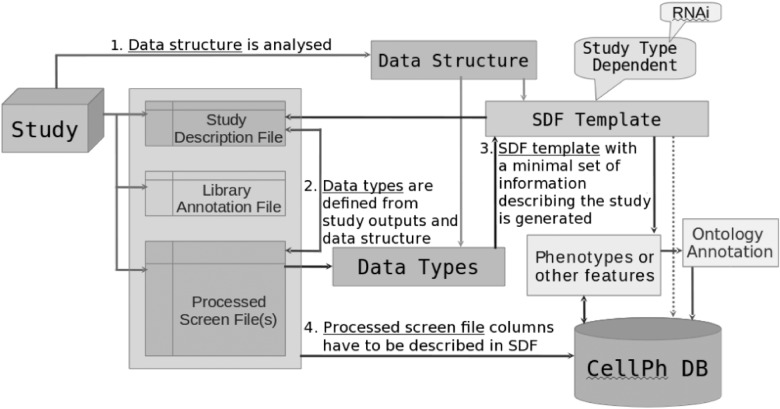
Data analysis and submission pipeline

CPD is built using MongoDB, an open-source document database engine, one of the most popular NoSQL databases (Stein, 2013). Data objects are stored in MongoDB collections, providing good scalability restricted only by the available storage capacity, as well as flexibility that enables extending the functionality of the system without major refactoring of the storage layers. This flexibility was very important during the system development phase, as well as for the potential future extensions (as discussed in the next section). The only drawback we can note is the availability of expertise that is important from the system support point of view, and currently is lower than for relational databases. Study data are loaded via a submission pipeline written in Perl. The data submission schema is shown in Figure 3

.

The web application is run by Mojolicious web server Hypnotoad, and was developed using Javascript library JQuery and Perl framework Mojolicious. Data in exported JSON format is also accessible through the public FTP (ftp://ftp.ebi.ac.uk/pub/databases/microarray/data/cellph/).

CPD does not provide facilities to manage image files or links to images, only processed, summary information. We believe that image archiving is a separate activity that requires building a separate resource.

As of time of writing the repository aggregates data from 10 independent RNAi studies. The total number of RNAi reagents from three suppliers is 93578; among these, 75568 have known sequences. We mapped these reagents onto the latest genome assemblies using bowtie (Langmead *et* *al.*, 2009) for Homo sapiens and bowtie2 (Langmead *et* *al.*, 2012) for *Drosophila melanogaster.*

Reagent mappings are visualized in the database interface, and only exact matches are taken into account. Every reagent that maps to multiple genes is shown with phenotype terms assigned, but in this case due to the off-target effect (Guest *et* *al.*, 2011) we cannot know which phenotypes are associated to which genes, therefore the corresponding genes do not have phenotypes displayed. 135 phenotype terms observed in the 10 studies are associated with 83 CMPO ontology terms.

Information from a phenotypic study is loaded into CPD from three separate data files:

1. Study Description File (SDF), which contains study and phenotype information. This file is divided into multiple sections. The ‘study metadata’ section contains study identifier, title, species, cell line, authors, results summary, specification of each study screen, i.e. protocol name and description.
   The ‘phenotype’ section contains phenotypes names, descriptions, information on whether the phenotype assignment has been done manually or automatically, names of phenotypes score parameters, logical expression describing the phenotype assignment rule (unless phenotypes have been directly assigned), and the scoring method.
   The ‘screen processed data’ section contains the processed data file name, checksum, name of each data column in the file as corresponds to its position, unit, description and data column types that are specific for each study type. See http://www.ebi.ac.uk/fg/sym/submit#examples for an example.
2. Reagents Library Annotation File (RLF) with sequences that are mandatory if we process an RNAi study, and other relevant reagent annotations if the study is not an RNAi experiment;
3. Processed Screen Files (PSF), the study output files with columns described in SDF. The number of these files has to be equal to the number of study screens (primary, validation). Each screen is described separately in the SDF.

In addition to the study metadata, i.e. study identifier, title etc. the SDF also contains instructions for automated submission pipeline (see Supplementary Appendix SI). These instructions help the submission processor to interpret and load the data directly from the study output files. See http://www.ebi.ac.uk/fg/sym/submit/ for more detailed information on the submission formatting requirements.

If a novel study type comes in (currently a non RNAi-based study), a new SDF template needs to be defined, which can then be completed directly by the data submitter.

CMPO terms and their mappings to the original phenotypic annotation are obtained from an external annotation process and can be updated on regular basis.

## 6 Discussion

CPD aims at providing easy access to high-throughput phenotypic data and integrates independent studies, adding significant value to the hard-earned primary data. Data integration in this domain is challenging for several reasons: (i) independent datasets are heterogeneous since they have been processed and analyzed using different pipelines; and (ii) systems microscopy has not achieved the same level of standardization as other ’omics’ domains, and no standards have been formally adopted for reporting, annotating and storing such data, making its representation more difficult.

To solve some of the issues associated with handling systems microscopy data, we have here proposed a minimal set of information for reporting system microscopy data, currently based on data derived from RNAi screens, but easily extendable to include other experimental methodologies.

In addition, CPD is the first resource to introduce use of a standardized ontology, CMPO, for querying and browsing cellular phenotypes, in an attempt to integrate phenotypic annotations from independent studies and enhancing the understanding of the phenotypes observed. CMPO coverage is still limited, and a larger volume of experimental data is needed before we can start uncovering interesting correlations between independent systems microscopy studies. CMPO development continues, and efforts to increase its coverage are underway. Additionally, the reproducibility between screens addressing the same biological process is limited due to many reasons, including differences in assays, siRNA libraries and statistical analysis pipelines utilized (Hancock, 2014).

Another resource that provides access to RNAi data is GenomeRNAi (Schmidt *et* *al.*, 2013). Compared to this database, CPD displays the gene-phenotype relationships in the form of heatmaps that facilitate data exploration, implements alternative ways to search and browse phenotypes including corresponding ontology-based terms, provides richer pages related to reagent-to-gene mapping information, as well as adds the possibility of browsing genes by the number of reagents mapped to them.

## 7 Conclusion

Although CPD has been established as a repository for systems microscopy data and currently contains only data processed from RNAi studies, its development has shown that the same software architecture paradigm can be used to manage other data related to genes, proteins, small molecules or any other molecular entities studied within a cellular context and which can have complex, not only binary, interrelationships. For instance, data from other kinds of cell perturbations such as drug screening could be managed within the CPD data model. Then, instead of ‘gene-phenotype’ relations, the database would store ‘drug-phenotype’ relations that could be browsed and queried in the same way as implemented for phenotypic data. Another example is localization studies where data is in the form ‘protein-intracellular object’ (see Fig 1).

The experience we had in developing CPD has highlighted the need for the systems microscopy community to introduce and use reporting standards and controlled vocabularies for data sharing and annotation. Such standards need to be agreed, developed and adopted by the systems microscopy community at large before data sharing in this field can be considered mature and comparable to other-omics data domains. Work on standards is ongoing within the Systems Microscopy Network of Excellence, and a report will be published before the end of 2015. We hope that the minimum reporting requirements, the data formats, and the ontology proposed here can become the foundation of a much larger harmonization effort in this domain.

## Supplementary Material

Supplementary Data
