# Supplementary material for: Cellular phenotype database: a repository for systems microscopy data
Source: Bioinformatics. 2015 Apr 9;31(16):2736–40. doi: 10.1093/bioinformatics/btv199 (PMC4528631; doi:10.1093/bioinformatics/btv199)
Supplement: Supplementary Data [file supp_31_16_2736__index.html]

Cellular Phenotype Database: a repository for systems microscopy data — Cellular phenotype database: a repository for systems microscopy data — Cellular phenotype database: a repository for systems microscopy data — Supplementary Data 

# Cellular phenotype database: a repository for systems microscopy data

## Supplementary Data

files

**Files in this Data Supplement:**

- Supplementary Data - zip file
